# Velvety tree ant extract is a chemotaxis repellent for *C. elegans*

**DOI:** 10.17912/micropub.biology.001531

**Published:** 2025-05-31

**Authors:** Malaya Gaerlan, Marco Carrillo, Sofia Ceva, Sowmya Chundi, Binta Diallo, Juliana N. Fong, Kelly Huang, Jennifer Jackson, Jasmine Padilla, Leslie Quintana, Katelyn Santa Maria, Sadie M. Sarkisian, Paloma R. Sequeira, Eva U. Tatlock, Penelope R. Baker, Luise Bachmann, Soyeon Park, Malia J. Perez, Mina E. Phipps, Shay Nair Sharma, Yvette Soto-Hernandez, Bryan H. Juarez, Cesar Mena, Griselda Morales, Mabel Gonzalez, Katherine Fiocca, Nicole Bradon, Max Madrzyk, Lauren A. O'Connell

**Affiliations:** 1 BIO161 Organismal Biology Lab, Stanford University, Stanford, California, United States; 2 Department of Biology, Stanford University, Stanford, California, United States

## Abstract

Ants use a range of compounds for interspecies interactions, but the neurogenetic mechanisms mediating these interactions are unclear. Here, we used chemotaxis assays with the nematode *Caenorhabditis elegans* to test if ant compounds can be detected by the worm nervous system and which chemosensory neurons are required for detection. We found that *C. elegans* avoid the extracts of velvety tree ants ( *Liometopum occidentale* ), and this response requires *osm-9* and *tax-4* positive neurons. These experiments were conducted by undergraduate students in an upper-division laboratory course, demonstrating how simple behavior assays conducted in a classroom setting can provide practical research experiences and new insights into interspecies interactions.

**Figure 1. Velvety tree ant ( Liometopum occidentale ) extract induces an osm-9 and tax-4 dependent repulsive chemotaxis response in Caenorhabditis elegans.**
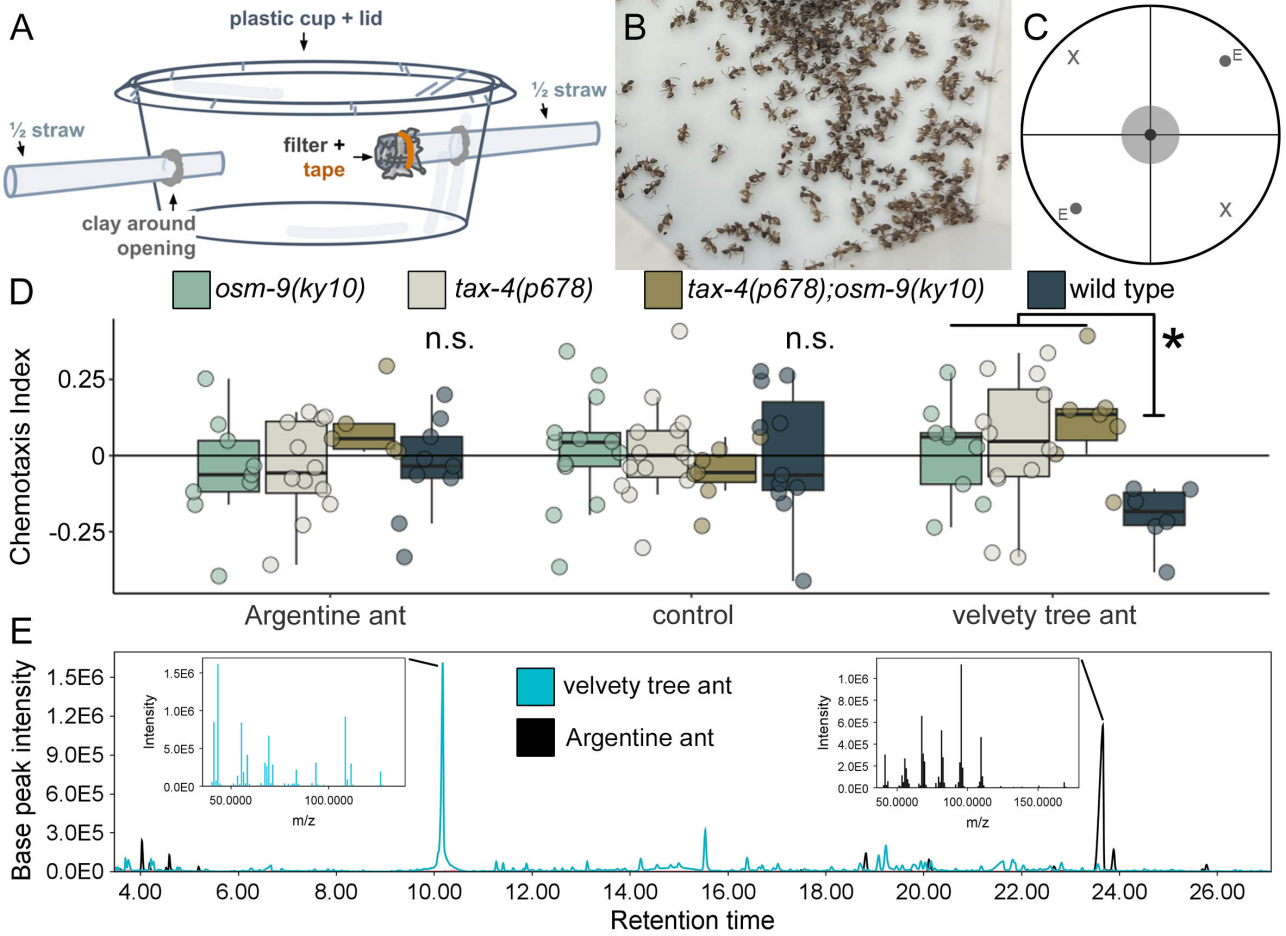
**(A)** A diagram of the aspirator students constructed to collect ants. **(B)** Ants collected and sorted by students on the Stanford University campus. **(C)** Chemotaxis assays were performed on circular plates divided into experimental (E) and solvent (X) quadrants. Worms were placed in the center. **(D)** The chemotaxis response of *osm-9(ky10)* knockout worms (green), *tax-4(p678)* knockout worms (beige), *tax-4(p678);osm-9(ky10)* double mutants (brown), and wild type worms (PD1074, dark blue) were tested in response to Argentine and velvety tree ant extracts. Asterisks indicate significant differences with a p-value below 0.05; n.s. (not significant) indicates comparisons with a p-value above 0.05. The number of assays per group are indicated as dots overlaid on the boxplots. **(E)** Representative total ion chromatogram of overlaid Argentine ant (black) and velvety tree ant (blue) chemical extracts. For velvety tree ants, the corresponding mass spectrum is included (insert) for the putative alarm pheromone detected at 10.17 min. For Argentine ants, the corresponding mass spectrum (insert) for iridomyrmecin, detected at 23.65 min, is included.

## Description

Ants are keystone organisms in many ecosystems and use chemicals to communicate within and between species (Fox and Adams, 2022; Jackson and Ratnieks, 2006; Parker and Kronauer, 2021). Chemical mediation of interspecies interactions are especially prominent in the context of invasions, where chemical secretions can be used by invasive species to kill competitors, while native species can use secretions to resist displacement (Greenberg et al., 2008; Sorrells et al., 2011). How ant-derived compounds interact with heterospecific nervous systems remains unclear due to the complexity of chemical extracts and the relatively limited genetic tools available for diverse ant species. Here, we utilized chemotaxis assays in the nematode worm *Caenorhabditis elegans* as a screening platform for ant-derived neuronal actuators. This genetic model organism has well-described chemosensory behavior, tractable genetics, and a mapped nervous system (Bargmann, 2006; Coburn and Bargmann, 1996), which enables relatively easy incorporation into undergraduate research experiences. We hypothesized that local ant species would contain compounds that interact with heterospecific nervous systems. We tested this hypothesis using (1) chemotaxis assays to identify ant extracts that interact with the *C. elegans* chemosensory system and (2) carried out these experiments in an undergraduate laboratory classroom that enables hands-on research experiences in chemical ecology.

We asked if extracts from Argentine ants ( *Linepithema humile* ) or velvety tree ants ( *Liometopum occidentale* ) influence *C. elegans* chemotaxis behavior and which chemosensory neurons are necessary for such a response. These ants are relatively abundant on the Stanford University campus (Vonshak and Gordon, 2015), allowing us to bridge field work with molecular assays in the laboratory. Chemosensory signaling in *C. elegans* can be ablated by removing specific cation channels like TAX-4 (Dusenbery et al., 1975; Komatsu et al., 1996) and OSM-9 (Colbert et al., 1997). TAX-4 is gated by intracellular cAMP levels whereas OSM-9 is responsive to osmotic, mechanical, and chemical signals. The *C. elegans* chemosensory neurons express one or both of these genes, making mutants useful for screening various natural products (Fryer et al., 2024), although these genes are also expressed elsewhere with different functions outside the chemosensory system. We tested the chemotaxis responses of *osm-9(ky10)* null mutants (Colbert et al., 1997), *tax-4(p678)* null mutants (Komatsu et al., 1996), *tax-4(p678);osm-9(ky10)* double mutants (Fryer et al., 2024), and wild type worms (PD1074) to ant extracts (Figure C) and found the worm strains responded differently (GLMM compound*strain: χ²(6) = 12.775, p = 0.047). Wild type (PD1074) worms were repulsed by velvety tree ant extracts, but mutant strains were not (wild type versus *osm-9* null mutant: t(101) = 2.524, p _adj_ = 0.026; wild type versus *tax-4* null mutant: t(101) = 2.915, p _adj_ = 0.013; wild type versus *osm-9* and *tax-4* double mutant: t(101) = 3.429, p _adj_ = 0.005), suggesting detection of velvety tree ant extracts requires *osm-9* and *tax-4* positive neurons. Worms did not respond to Argentine ant extracts or solvent control.

Our findings demonstrate that *C. elegans* can detect and respond to ant chemical extracts. Specifically, we found that *C. elegans* is repulsed by velvety tree ant extracts, which is consistent with a previous study (Alfonso et al., 2023), and that this response requires TAX-4 and OSM-9 ion channels. It is unclear which chemosensory neurons are responsible for this effect, and additional mutant screens or cell ablations would be required to pinpoint the neurons important for detection. Moreover, while this response requires TAX-4 and OSM-9, ant compounds are likely detected by G protein coupled receptors, and additional mutant screens targeting this protein class are required to identify proteins responsible for detection. The responsiveness of *C. elegans* to Argentine ant extracts is variable across studies. In this study, we did not observe a response to extracts of Argentine ants, which is consistent with one previous study (Lopez et al., 2024). However, this finding is inconsistent with another (Alfonso et al., 2023), which found *C. elegans* are repelled by Argentine ant extracts and this response is mediated by OSM-9. These variable responses may be due to concentration of the compounds or fluctuating chemical profiles across colonies or collection years (Nangia et al., 2025; Salado et al., 2023; Villalta et al., 2020). Additional experiments of varying stimulus concentrations, chemical sampling of more ant species and collection sites, as well as using purified ant compounds in chemotaxis assays would be required to test these alternatives.

To better understand the chemical composition of the ant extracts used in chemotaxis assays, we used gas chromatography / mass spectrometry (GC/MS) to tentatively identify ant compounds. Within the velvety tree ant extract, we detected and identified 6-methyl-5-hepten-2-one at 10.17 min (Figure E). This compound, also known as sulcatone, is used as an alarm pheromone in some ant species (Hoey-Chamberlain et al., 2013; Naragon et al., 2022; Stoeffler et al., 2007), including within *Liometopum (Casnati et al., 1964)* , but was not detected in *L. humile* . Within the Argentine ant extract, we detected iridomyrmecin at 23.65 min ( Figure 1 E), the main component of the Argentine ant defense compound (Alvarez-Blanco et al., 2021; Salado et al., 2023; Welzel et al., 2018). Although it is unknown which metabolite, combination of metabolites, or chemical concentrations are responsible for the observed response to velvety tree ant extract, sulcatone is known to elicit a response in other organisms beyond ants and is used by many insects in chemical communication (Malcicka et al., 2015; Schiestl, 2010; Stoeffler et al., 2007).

In summary, our study shows that velvety tree extract is a chemotaxis repellent for *C. elegans* , and this response requires *osm-9-* and *tax-4* -positive chemosensory neurons. Future studies including additional mutant strains would provide other mechanistic information on the intracellular signaling pathways responsible. Additional chemotaxis assays with a purified extract of the putative alarm pheromone that was identified in velvety tree ant extracts would reveal if this compound is responsible for the behavioral effect reported here. Finally, this research, along with previous studies by students in this course over multiple years (Alfonso et al., 2023; Lopez et al., 2024), has highlighted the value of using simple behavior assays in undergraduate research classrooms to make original scientific discoveries through genuine research experiences and the value of longer-term experiments in understanding the dynamics of chemical ecology over time.

## Methods

*Worm strains*

Nematode strains ( *Caenorhabditis elegans* ) were obtained from the *Caenorhabditis* Genetics Center (CGC) at the University of Minnesota or from the lab of Miriam Goodman at Stanford University. Animals were maintained in 20℃ incubators and synchronized by bleaching adults to obtain eggs. Eggs were grown on nematode growth media plates spread with OP50 *Escherichia coli* , as described elsewhere (Stiernagle, 2006). Hatched eggs were kept at 20℃ for 2-5 days, depending on the strain, when the population reached a young adult stage (L4) and were used for chemotaxis assays.

*Ant extracts*

Students built aspirators using plastic condiment cups, plastic straws, clay putty, and strips of panty hose (Bradon et al., 2023). These aspirators were used to collect workers of both Argentine ants ( *Linepithema humile* ) and velvety tree ants ( *Liometopum occidentale* ) from trees on the Stanford University campus. Ants were incubated at -80℃ for 15 min, and then students sorted the ants from debris and placed them into glass vials of methanol (1:1 methanol:ant volume). These vials were incubated at -80℃ for 24 hours before moving the methanol to a new glass vial. An aliquot was saved for chemical analysis (see below), and the remaining methanol was evaporated under nitrogen gas. The remaining ant extract residue was resuspended in 1:1 dimethyl sulfoxide (DMSO):ant volume for use in chemotaxis assays.

*Chemotaxis assays*

Chemotaxis assays were conducted by undergraduate students as previously described (Alfonso et al., 2023; Bradon et al., 2023; Lopez et al., 2024). Students were unaware of the compound or strain being tested until data was submitted to the instructor. Chemotaxis plates [5mM KPO4 (pH 6), 1mM CaCl2, 1mM MgSO4, 2% agar] were divided into four quadrants (Figure C) and 5 μL ant extract (concentration unknown) were placed on dots located in two non-adjacent quadrants (E, experimental), while 5 μL of DMSO (solvent) was placed on X marks in the other two non-adjacent quadrants. Solvent was also placed in the experimental quadrants of the control plates, meaning all four quadrants contained DMSO. Plates were incubated for 30 min. During this time, students prepared the worms for chemotaxis assays by removing them from the growth plates using chemotaxis assay buffer [5mM KPO4 (pH 6), 1mM CaCl2, 1mM MgSO4]. The worms were washed two to three times until the liquid became clear from debris as the worms settled at the bottom of the tube. After compound incubation but before worms were placed on the chemotaxis plates, 2 μL 0.5 M sodium azide solution was applied to each of the quadrant spots to paralyze the worms at those locations. Then, roughly 100 worms were placed in the center of each chemotaxis plate and allowed to roam for 30 min. Worms were counted manually under a dissecting microscope using a tally counter. Worms in the center circle area were not counted to avoid including dead worms in the dataset.

*Gas chromatography / mass spectrometry analysis*

All ant samples were analyzed via gas chromatography coupled to mass spectrometry (GC/MS). The system used included a GC-2030 NEXIS, a single quadrupole mass spectrometer (QP-2020), and an autosampler SPL AOC-20i (Shimadzu, Nakagyo-ku, Kyoto, Japan). From each ant extract, 1 µL of methanol was injected at 250°C in splitless mode, and the separation occurred on an HP-5MS capillary GC column (30 m x 0.5 mm x 0.25 µm, Agilent, Palo Alto, CA, USA) at a flow rate of 1.0 mL/min. The temperature program began at 40℃ for 3 min, increased to 100℃ at a rate of 6℃/min, then to 200℃ at 4℃/min, and finally to 300℃ at a rate of 20℃/min where it was maintained for 3 min. The ion source was set at 150℃, and the interface temperature was set to 230℃. The mass spectrometer was operated in full scan mode and performed a scan every 0.2 s, using a mass range of 40-500 m/z. The resulting GC/MS files were converted to .CDF and opened in MZmine3 (Schmid et al., 2023) to create the plot in panel E. Putative annotations of the suspected velvety tree ant alarm pheromone and Argentine ant defense compound (iridomyrmecin) were performed using GCMS Postrun Analysis software (Shimadzu) using NIST 14 and Wiley databases.

*Data analysis*

The Chemotaxis Index (CI) was calculated for each plate, where CI = (Number of worms in the two experimental quadrants – Number of worms in the two solvent quadrants) / Total number of worms. Roughly the same amount of assays were performed for each experimental group. Plates were removed from the dataset if they had less than 30 or more than 300 worms to reduce large differences in worm abundance and to avoid statistical artifacts related to inter-experimenter differences in their ability to transfer or count roughly 100 worms. Plates were also removed if a student noted a technical error in the plate setup, such as mistakes in the placement of worms or compounds. After this processing step, the number of assays per group were as follows for Argentine ants, solvent, and velvety tree ants, respectively: *osm-9(ky10)* knockout N = 9, 13, 8; *tax-4(p678)* knockout N = 12, 14, 12; *tax-4(p678);osm-9(ky10)* double mutants N = 5, 6, 7; wild type PD1074 N = 9, 11, 6. The number of assays for double mutants is lower because this worm strain grows much slower and plates contained fewer worms at the start of class, leading to fewer worms in the chemotaxis assays and a higher probability of exclusion.

Data analysis and visualization were performed in R version 4.4.1 (R Core Team, 2024) in RStudio version 2023.03.1+446. We used the glmmTMB package version 1.1.7 (Brooks et al., 2017) for statistical analyses using generalized linear mixed models and followed the model with the Anova.glmmTMB function using Type III “marginal” sums of squares for reported statistical values. Appropriate model diagnostics were confirmed with the DHARMa package version 0.4.6 (Hartig, 2024). The number of worms in the experimental quadrants divided by the total number of worms on the plate was used as the dependent variable, while compound, worm strain, and their interaction were the independent variables. Post hoc analyses were performed using emmeans version 1.8.6 (Lenth, 2024) with false discovery rate (fdr) adjustment of p-values to account for multiple comparisons.

The drawing of the ant aspirator was created in the Notability App (version 14.10.4) on an iPad. The photo of ants in Figure panel B was taken by a student using a phone camera. Boxplots were generated using the ggplot2 version 3.4.3 (Wickham, 2016) package.

*Classroom pedagogy*

The experiments in this study were performed over two laboratory class sessions. These sessions were preceded with one training session where students learned how to conduct *C. elegans* chemotaxis assays using known attractant and repulsive compounds. One laboratory session involved collecting ants on the Stanford University campus using aspirators constructed by the students. The ant extracts students collected were used to conduct chemotaxis assays in another classroom session. An additional classroom session not reported here was used to introduce mass spectrometry methods. Additional chemotaxis assays were carried out by the teaching staff on a different day to validate the chemotaxis results. Weekly homework included reading relevant literature, analysis and visualization of data collected by the entire class, and writing individual drafts of a journal-style article, which were combined into this article by the instructors. Assignments were graded as complete/incomplete, and students received detailed feedback at each stage. All students edited and approved of the initial and revised manuscripts.

## Reagents

**Table:** 

**Strain**	**Genotype**	**Source**
PD1074	Wild type	Caenorhabditis Genetics Center (CGC) at the University of Minnesota
PR678	*tax-4 ( p678 )* III	Caenorhabditis Genetics Center (CGC) at the University of Minnesota
CX10	*osm-9 ( ky10 )* IV	Caenorhabditis Genetics Center (CGC) at the University of Minnesota
GN1077	*tax-4 ( p678 )* III; *osm-9 ( ky10 )* IV	Miriam Goodman Lab at Stanford University
